# Case of Tape-worm Occurring in Connection with the Eating of Raw Pork

**Published:** 1856-04

**Authors:** 


*Case of Tape-worm occurring in connection with the Eating of Raw Pork.*—At the Medico-Chirurgical Society of Edinburgh, Dr. Gairdner narrated the case of a girl, at present under his care in the Infirmary, which seemed to support the views of Siebold and Kuchenmeister, as to the transformation of the cysticercus cellulosæ, found in the hog and other domestic animals, into the *tænia solium.* Nine yards of the tape-worm had been expelled under the action of the shield-fern oil. On inquiry, the girl admitted that she had been in the habit of eating quantities of raw pork and butcher-meat generally. This was from a peculiar liking or inclination of her own, and was not a habit contracted in consequence of the example of others. In other respects her diet had been similar to that generally in use in her station in life in Scotland. It was well ascertained, that in Scotland the occurrence of tape-worm was rare as compared with some parts of England, and very rare when compared with some European countries. It was not less unusual in Scotland to indulge in the eating of raw flesh, which practice was believed to be a frequent source of the production of tænia. The occurrence of tænia was very common in Germany, where the practice of eating raw ham was also prevalent. On the other hand, Dr. G. had reason to believe that tænia was rare in Holland, where the eating of raw animal food is very unusual. Dr. G. alluded to a case lately published by Dr. Crighton (*Monthly Journal,* June, 1855,) in which he had been able to trace the occurrence of tænia solium to the practice of eating raw meat, a practice which was common among the Lancashire operatives. Dr. G. was inclined to attribute the rarity of the occurrence of hydatids in Scotland, to the small proportion of animal food, and especially of ill-cooked animal food, used by the laboring classes. During Dr. G.'s connection, as pathologist, with the Infirmary, he had opened not fewer than 1500 bodies, and he had never met with a single case of hydatids of the liver. Two cases had otherwise come under his notice; but in his dissections at the Infirmary, he had never seen one instance of the occurrence of the acephalocyst. In the London hospitals a considerable number were known to occur every year.—*Edinburgh Med. Journ.*

